# Does time heal all wounds? Examining the lasting impact of historic redlining on contemporary racial disparities in allostatic load in Baltimore, Maryland

**DOI:** 10.1080/13557858.2026.2635109

**Published:** 2026-03-08

**Authors:** Marcus R. Andrews, Linda M. Chatters, Roshanak Mehdipanah, Andrew Grogan-Kaylor, Michele K. Evans, Alan B. Zonderman, Tiffany M. Powell-Wiley, Amy J. Schulz

**Affiliations:** a Department of Health Behavior and Health Education, University of Michigan School of Public Health, Ann Arbor, MI, USA; b School of Social Work, University of Michigan, Ann Arbor, MI, USA; c Laboratory of Epidemiology and Population Sciences, National Institute on Aging, National Institutes of Health, Baltimore, MD, USA; d Social Determinants of Obesity and Cardiovascular Risk Laboratory, National Heart, Lung, and Blood Institute, National Institutes of Health, Bethesda, MD, USA; e Intramural Research Program, National Institute on Minority Health Disparities, National Institutes of Health, Bethesda, MD, USA

**Keywords:** Allostatic load, neighborhood environment, HOLC score, health disparities, urban health, Good health and wellbeing, reduced inequalities

## Abstract

**Objective:**

To examine associations between historical residential redlining, a form of institutional racism, and contemporary allostatic load (AL), an indicator of physiologic stress, examine differences by race, and whether associations are mediated by neighborhood deprivation.

**Methods:**

We used data from Wave 4 (2013–2017) of the Healthy Aging in Neighborhoods of Diversity across the Life Span (HANDLS) study in Baltimore, MD, Home Owners Loan Corporation (HOLC) scores, and data from the American Community Survey (ACS). We conducted a multilevel analysis, controlling for individual demographic and behavioral characteristics.

**Results:**

Compared to blue– and greenlined neighborhoods, living in a yellowlined neighborhood was positively associated with allostatic load (*p* < 0.05). We report a significant negative interaction between living in a yellowlined area and race (*β* = −0.62, *p* = 0.00), with African American residents showing greater reductions in allostatic load compared with whites. We also found evidence of a moderated mediation, with the indirect effect of living in a yellowlined area on AL through NDI varying by race.

**Conclusions:**

Contemporary residents of historically yellowlined, but not redlined, neighborhoods experienced heightened physiological deterioration. The strength of this association differed by race, and was substantially explained by the contemporary neighborhood deprivation index only for African Americans in yellowlined areas. Addressing health disparities requires attention to the long-lasting impacts of historical practices of institutional racism on contemporary health outcomes.

## Introduction

Allostatic load (AL), the cumulative ‘wear and tear’ of stress on physiological systems, has been associated with a series of adverse health outcomes, including cardiovascular diseases (Nelson et al. 2007; Sabbah et al. 2008), major cardiac events (Stabellini et al. 2024), and all-cause mortality (Parker et al. 2022). Specifically, allostatic load measures heightened physiological responses associated with persistent exposure to stressful environmental stimuli (Guidi et al. 2020; McEwen and Stellar 1993). During stressful events, the body releases physiological mediators (e.g. cortisol, epinephrine, dopamine, and serum dehydroepiandrosterone sulfate) (Beckie 2012; Dowd, Simanek, and Aiello 2009), that are associated with changes in blood pressure, HDL cholesterol concentrations, total-cholesterol-to-HDL-ratio, and heart rate, factors that are known predictors of cardiovascular risk (Dowd, Simanek, and Aiello 2009; Gillespie et al. 2019; McEwen and Seeman 1999).

Recent evidence indicates that higher allostatic load, and associated risks, are not evenly distributed within the United States; however, findings are mixed. For example, some literature reports higher allostatic load among African American adults (Andrzejak et al. 2023; Bey et al. 2018; Chen et al. 2014; Chyu and Upchurch 2011; Chyu and Upchurch 2018; Cobb et al. 2016; Kezios et al. 2022; Moore et al. 2022; Nobel et al. 2017; Obeng-Gyasi et al. 2023; Peek et al. 2010; Stabellini et al. 2024; Walsemann, Pearson, and Abbruzzi 2022), while others report higher allostatic load scores among white adults (Beydoun et al. 2022) or no differences by race (Brooks et al. 2014; Salinas and Sexton 2015). These inconsistencies highlight the need for further investigations into associations between potential differences in allostatic load by race. The mixed evidence may be attributable to differences in the biomarkers used to measure allostatic load, the geographic context of the study cohorts, and whether the analyses were conducted with national or local samples.

Racial residential segregation, one manifestation of institutional racism, has had profound implications for neighborhood contexts across the United States (Winling and Michney 2021). Specifically, racial residential segregation shapes differences in socioeconomic status, thus impacting educational and employment opportunities (Williams and Collins 2001), which are known protective factors for health (Bonnie, Stroud, and Breiner 2015). One of the more consistent factors shaping historical discriminatory housing practices came in the form of redlining. ‘Redlining’, one of the mechanisms through which institutional racism has been enacted, involved appraising neighborhood characteristics and ranking them based on presumed lending risk (Winling and Michney 2021). The term was coined from the Home Owners’ Loan Corporation’s (HOLC) residential security maps and appraisals of neighborhoods during President Roosevelt’s attempt to stabilize the United States economy during the Great Depression and New Deal era of the 1930s (Greer 2013). Local bankers and other real estate actors appraised and classified neighborhoods across the country into one of four categories: the most desirable neighborhoods were labeled ‘A’ and were shaded green, and the least desirable neighborhoods were labeled ‘D’ and shaded red (Nardone et al. 2020). In addition to racial composition, HOLC grades assessed infrastructure, housing quality, neighborhood stability, and proximity to different hazards (Winling and Michney 2021). ‘Redlining’ refers specifically to those neighborhoods labeled ‘D’ and shaded red. Given that the HOLC had already issued most of its loans before the HOLC maps were created, HOLC maps did, however, assess the risks of already-issued loans and helped manage and resell foreclosed housing stock (Fishback et al. 2024; Greer 2013; Hillier 2003; Michney and Winling 2020). While lesser preserved, due to the destruction of housing grade maps, the actions of the Federal Housing Administration (FHA) must also be taken into account (Fishback et al. 2024). Unlike the HOLC, the FHA used ratings from its inception to determine risk based on racial discrimination (Fishback et al. 2024). Risk was assessed based on the deterioration of physical structures within an area as well as the likelihood of neighborhood demographic transition (Fishback et al. 2024). Drawing upon the fear that the entrance of African American populations into white neighborhoods would decrease property values, the FHA would not insure loans in neighborhoods where racial demographics could change (Akbar et al. 2019).

While HOLC maps are the most widely preserved maps of historical housing discrimination, they were not the primary federal tool for maintaining racial residential segregation. The Federal Housing Administration used racial and ethnic composition to determine lending risk to prevent areas from being integrated (Fishback et al. 2024). Therefore, HOLC maps should be understood more as an indicator of localized neighborhood stigma and the racial attitudes already in place.

Winling and Michney found that while not all redlined neighborhoods were predominantly African American, nearly all predominantly African American neighborhoods were redlined (Winling and Michney 2021). Immigrant groups, including people of Mexican, Chinese, Russian, Italian, and Polish descent, were largely concentrated in yellow – and redlined neighborhoods (Madron 2023). Although formal redlining was outlawed in the United States with the passage of the Fair Housing Act of 1968, its impacts on neighborhoods labeled ‘hazardous’ continue, and many neighborhoods remain under-resourced (Aaronson, Hartley, and Mazumder 2020; Mitchell and Franco 2018). Currently, many neighborhoods that were designated as yellow (declining) or redlined (hazardous) remain lower-income communities of color, while areas assessed as ‘desirable’ typically remain predominantly white, higher-income communities (Aaronson, Hartley, and Mazumder 2020; Mitchell and Franco 2018; Nardone et al. 2020).

Contemporary census tract-level data about neighborhoods scored using the HOLC system are publicly available through the University of Richmond’s Mapping Inequality Project (Nelson et al. 2018). With that newly available data, emerging research connects historical redlining to current neighborhood health characteristics, including asthma (Nardone et al. 2020), maternal and child health outcomes (Hollenbach et al. 2021; Huang and Sehgai 2022; Krieger et al. 2020; Lynch et al. 2021; Nardone et al. 2020), self-rated health (McClure et al. 2019), rates for mortality 41, cancer (Krieger et al. 2020), cardiovascular risk (Mujahid et al. 2021; Nardone et al. 2020), COVID-19 (Li and Yuan 2022), and emergency department visits/hospitalizations (Benns et al. 2020; Li et al. 2022). Redlining has also been linked to current neighborhood socioeconomic disadvantage (Mitchell and Franco 2018), sustained disinvestment (Andrews et al. 2025), lending discrimination (Lynch et al. 2021), and reduced access to health-promoting resources (Mehdipanah, McVay, and Schulz 2023). Specifically, a recent study examined whether neighborhood structural investment mediates ecological associations between HOLC grade and cardiometabolic indicators (obesity, diabetes, and coronary heart disease prevalence) across 16 U.S. cities (Andrews et al. 2025). They reported that living in a redlined neighborhood was negatively associated with neighborhood investment in many of these cities, and that higher investment, in turn, was negatively associated with cardiometabolic indicators. Taken together, these findings that neighborhood investment predicts lower cardiometabolic disease prevalence, alongside the findings that report that neighborhood economic deprivation positively associates with allostatic load (Ribeiro et al. 2019; Schulz et al. 2012; Seeman et al. 2008), highlights that neighborhood economic contexts may be a potential pathway through which historical redlining impacts contemporary health outcomes.

Despite evidence of its associations with health outcomes, the relationship between HOLC scores and allostatic load, as well as potential mediators, remains understudied. Studies of allostatic load present a unique opportunity to potentially understand the different symptomology profiles seen in clinical practice, before actual progression to disease states. In a disease-centered medical system, these physiological indicators are often overlooked (Guidi et al. 2020; Henningsen et al. 2018; Kroenke 2014). Race-based residential segregation and the associated social and environmental consequences of redlining are a fundamental cause of health because it is linked to multiple health outcomes through multiple pathways (Williams and Collins 2001). Consistent with the Fundamental Cause theoretical framework, redlining shapes racial health inequities as it ‘concentrates risks and limits opportunities for communities of color’ (Williams and Collins 2001), p. 110] Understanding and addressing contemporary racial inequities in health requires examination of historical redlining and disinvestment processes and their implications for the creation and continuance of racial health inequalities, including allostatic load.

### The case for Baltimore, Maryland

Baltimore remains highly geographically segregated, with African American residents concentrated in the eastern and western halves of the city and high rates of poverty concentrated in predominantly African American neighborhoods (Brown 2016; Theodos, Hangen, and Meizell 2019). This study explores associations between former redlining practices, contemporary neighborhood characteristics, and allostatic load within a local cohort. This specific and local focus can elucidate pathways and processes that reveal heterogeneity in local lived experiences that are difficult to discern when using data from nationally representative samples.

## Specific aims and hypotheses

Given that people with different social positions (e.g. race, gender) living in the same neighborhood may experience neighborhood conditions differently (Millar 2020; Velasquez et al. 2022), and considering evidence that redlining is associated with both contemporary health outcomes and economic conditions in tandem with racial differences in allostatic load, we examine whether:

Q1: Historical HOLC scores associated with allostatic load scores among current (2013–2017) residents of Baltimore, MD, and if these associations vary by race.

Q2: The relationship between historical HOLC scores and allostatic load scores is mediated by neighborhood deprivation.

We hypothesize that historical HOLC scores will be positively associated with contemporary allostatic load and that these associations will vary by race. We also hypothesize that the relationship between HOLC score and allostatic load will be mediated by neighborhood deprivation/poverty.

## Methods

The Healthy Aging in Neighborhoods of Diversity across the Life Span (HANDLS) study is based on a cohort of 3,720 white and African American residents from socioeconomically diverse communities in Baltimore, who were between 30 and 64 years old at baseline (2004–2009). Participants are from a fixed cohort from 13 neighborhoods based on area probability sampling. Other study design and methodology-related materials for HANDLS have been published in detail elsewhere (Akbar et al. 2019). The analytic sample for this study includes participants who: (a) lived in Baltimore during Wave 4 (2013–2017) of HANDLS; (b) have an allostatic load measurement; (c) live in a census tract, within the city limits of Baltimore, where historical HOLC scores were calculated; and (d) have an observation for each included covariate. This study was approved by the University of Michigan (HUM00211850) and the National Institute of Aging IRB.

### Independent variable: redlining (1930s)

Historical HOLC score data were downloaded from the University of Richmond’s Mapping Inequality dataset, which provides HOLC score grading normalized to 2010 census tracts across many U.S. cities (Nelson et al. 2018; Meier and Mitchell 2021). Greater detail about the construction of the HOLC scores in this dataset is available (Nelson et al. 2018). Scores range from 1 (greenlined) to 4 (redlined). Due to fewer respondents living in green- and bluelined census tracts, green- and bluelined census tracts in the HANDLS dataset used for this study, green- and bluelined census tracts were combined into a single category.

### Mediator: neighborhood deprivation

We also test whether neighborhood deprivation index (NDI), measured at the census tract level, mediates associations between HOLC score and AL. NDI was calculated using previously published methods drawn from the 2017 (5-Year Estimates) American Community Survey to align with Wave 4 of the HANDLS data collection (Powell-Wiley et al. 2020). Variables used to calculate this composite index include median household income, median housing value, the percentage of individuals over 25 with a high-school diploma, the percentage of individuals over 25 with a bachelor’s degree, the percentage of employed people (age 16 and older) working in management, business, science, and arts, the percentage of households below the federal poverty limit, the percentage of families receiving public assistance, the percentage of female-headed households with children under 18, and the percentage of people that receive interest, dividends, or rental income.

### Dependent variable: allostatic load

Allostatic load is a composite index computed using methods described by Seeman and colleagues (Seeman et al. 2008). Components of this index include cardiovascular (systolic and diastolic blood pressure, pulse rate), metabolic (total cholesterol, high density lipoprotein-cholesterol [HDL], glycated hemoglobin, waist-to-hip ratio, and inflammatory (albumin, C-Reactive Protein [CRP]) indicators (see Mitchell and Franco (2018) for additional descriptions of these component variables). Risk scores were assigned based on previous research, where a score was given based on clinical cut points (Beydoun et al. 2019). Specifically, high risk was defined by having a(n) albumin <3.8, C-reactive protein ≥0.3, Waist:Hip >0.9 for men; 0.85 for women, Total cholesterol ≥240, HDL <40, Glycated hemoglobin ≥6.4, Resting heart rate ≥90, Systolic blood pressure, ≥140, and Diastolic blood pressure ≥90. These clinical cut points and specific methods to measure allostatic load follow protocols that have been extensively reported in the literature (Evans et al. 2025).

### Covariates

Informed by similar research investigations (Ribeiro et al. 2019; Schulz et al. 2012), the following covariates were included: age (35–44 45–54, 55–64, 65–76), race (white vs. African American), sex assigned at birth (female vs. male), physical activity (measured by the question ‘How many minutes per day do you walk or cycle to and from work or shopping?’ < 5 min 5–15 min, 15–30 min, 30–45 min, >45 min), current tobacco smoking status (never tried or never used regularly, former user, current user), and household poverty status (self-reported household income based on 125% of the 2004 Health and Human Services Poverty Guidelines <125% = below poverty; PIR ≥125% = above poverty).

### Statistical analysis

StataSE 17 (StataCorp, College Station, TX, USA) was used to conduct all analyses. We calculated descriptive statistics for all variables included in the analyses. Since each participant constituted one observation and multiple observations were clustered in a census tract, some of the characteristics of the individual could be correlated with the characteristics of others within the cluster (Rose 2018). Accordingly, a multilevel modeling approach for both crude and adjusted analyses, specifying random intercepts at the census tract level to account for between-tract variation in allostatic load while estimating the fixed effects for both individual- and tract-level indicators. We also include a cross-level interaction between HOLC score and individual-level race to test whether the effect of HOLC score on allostatic load varies by race. To examine potential mediating pathways, we conducted structural equation modeling, including the interaction between race and HOLC score, where indirect effects were tested using bootstrapping with 5,000 replications to provide more accurate confidence intervals for mediation effects. Statistical significance was assessed when *p* < 0.05.

## Results

A total of 1,013 participants had measures for the main exposure, outcome, and covariates for this study. Table 1 presents the descriptive statistics for each of the variables included in the study. The mean allostatic load score for study participants was 1.95 (min = 1, max = 6); 30.95% lived in formerly green or bluelined, 55.84% in yellowlined, and 13.21% in redlined census tracts. Approximately 11.21% of the sample was between 35 and 44 years old, 30.01% was between 45 and 54 years old, 36.81% was between 55 and 64 years old, and 21.97% between 65 and 76 years old. Nearly 70% of the sample was African American, and nearly 60% of the sample was female. For physical activity, 31.69% of participants reported getting less than 5 min per day, while 23.49% indicated 15–30 min, 20.73% indicated 5–15 min, 13.13% indicated more than 45 min, and 10.96% indicated 30–45 min per day. Smoking status varied, with 42.74% of study participants being current users, 25.67% former users, 20.14% never tried, and 11.45% tried but never used regularly. Six out of ten (62.69%) participants had household incomes above the poverty threshold, while 37.31% were at or below this level.

A total of 138 census tracts were included in the analytic sample, with an average of 7 participants per census tract, and 115 census tracts with more than 10 participants in them. Further, when looking at allostatic load trends by race and HOLC category, whites, compared to African Americans, had lower allostatic load scores in the Greenlined and Bluelined groups as well as the redlined groups (Table 1). However, whites had higher allostatic load scores than African Americans in the Yellowlined areas.

### Are HOLC scores associated with allostatic load?

Compared to green- and bluelined areas, living in a yellowlined area was positively associated with AL, but not in redlined areas (*β* = 0.18, *p* = 0.03, *β* = 0.18, *p* = 0.16, respectively, Table 2, Model 1). These relationships remained after adjusting for age group, race, sex, physical activity, smoking status, and household poverty status (*β* = 0.21, *p* = 0.01; *β* = 0.23, *p* = 0.08; Table 2, Model 3).

### Are there racial differences in the association between HOLC score and AL?

We report a significant interaction in the associations between allostatic load and residence in a formerly yellowlined area and African American race (*β* = −0.62, *p* = 0.00; Table 2, Model 3), whereas the corresponding interaction between residence in a formerly redlined area and African American race was not significant (*β* = −0.34, *p* = 0.28, Model 3) in this sample. The interaction term suggests that the effect of living in a yellowlined, compared with blue – or greenlined area, on allostatic load is smaller for African Americans compared to whites.

### Does neighborhood deprivation index mediate associations between HOLC and allostatic load?

Given racial differences in the association between HOLC score and allostatic load, we test whether race also moderates the potential mediating effect of neighborhood deprivation index (NDI) on associations between HOLC score and allostatic load. Findings reported in Table 3 indicate that living in a yellowlined area in 2017 was positively associated with NDI relative to green – and bluelined areas (*β* = 0.80, *p* = 0.00); living in redlined areas trended in the expected direction but did not reach statistical significance at the *p* < 0.05 level (*β* = −0.65, *p* = 0.08). The association between redlined areas and NDI differed by race (*β* = 0.48, *p* = 0.02); racial differences in associations between yellowlined areas and NDI were not significant (*β* = 0.01, *p* = 0.92) (path a). NDI is positively associated with allostatic load (*β* = 0.41, *p* = 0.01), and this association was moderated by race (*β* = −0.05, *p* = 0.047). Bootstrapping analyses report evidence of a moderated mediation for yellowlined areas on allostatic load through NDI was only significant for African Americans in this area (*β* = 0.29, *p* = 0.04), but not for white respondents (*β* = 0.33, *p* = 0.07). Further, there were no indirect effects of redlined areas on allostatic load through NDI for any racial group (*β* = −0.27, *p* = 0.28; *β* = −0.06, *p* = 0.55).

## Discussion

This study investigated the relationship between historical redlining and contemporary allostatic load and tested whether these associations were moderated by race and mediated by a neighborhood deprivation index. Findings are consistent with the hypothesis that historical HOLC scores are associated with contemporary allostatic load scores, though these associations are weaker for African Americans than for whites. Test of moderated mediation effects of neighborhood deprivation on associations between HOLC category and allostatic load reports a significant mediation effect among African Americans living in yellowlined areas, but not for African Americans in redlined areas or for whites in either yellow – or redlined areas.

### HOLC score and allostatic load

After accounting for social and behavioral characteristics of individuals, we found that contemporary residents of census tracts historically designated as yellowlined had significantly higher allostatic load scores compared with those living in formerly blue or greenlined areas. Though no previous study of which we are aware has examined associations between HOLC score and allostatic load, our findings are congruent with several studies that have found a positive association between HOLC score and cardiovascular indicators (Andrews et al. 2025; Mujahid et al. 2021; Nardone et al. 2020; White, Guikema, and Logan 2021). Thus, our study adds to the empirical support between historical redlining and contemporary adverse health outcomes while also supporting ‘fundamental cause’ theoretical frameworks that link historical policies that institutionalized racism with contemporary health status through their impacts on physiological responses to stress (Greer 2013).

### Race differences in associations between HOLC and allostatic load

Findings reported here regarding associations between HOLC score, race, and allostatic load are partially consistent with findings reported elsewhere in the literature (Andrzejak et al. 2023; Bey et al. 2018; Chen et al. 2014; Chyu and Upchurch 2011; Chyu and Upchurch 2018; Cobb et al. 2016; Kezios et al. 2022; Moore et al. 2022; Nobel et al. 2017; Obeng-Gyasi et al. 2023; Peek et al. 2010; Stabellini et al. 2024; Walsemann, Pearson, and Abbruzzi 2022). Specifically, our descriptive findings show higher levels of allostatic load among residents of formerly yellowlined and redlined neighborhoods, compared to those living in formerly blue – and greenlined neighborhoods. Similarly, African Americans reported higher levels of allostatic load compared with white participants in this study, again consistent with previous literature.

The results from multilevel regression models reported significant differences by race for the association between HOLC score and allostatic load. Specifically, while white residents of formerly yellowlined areas had higher allostatic load scores compared to whites living in formerly blue – and greenlined areas, we did not observe comparable associations for African American residents of yellowlined areas. We did not find differences by race in associations between HOLC score and allostatic load in redlined areas. These findings differ somewhat from results reported by Mujahid et al. (2021), who examined race-specific associations between historical redlining scores and cardiovascular health in a multi-city sample (Chyu and Upchurch 2018). Their findings indicated that associations between HOLC category and ideal cardiovascular health were only significant among African Americans but not Hispanic, Chinese, or white participants in the Multi-Ethnic Study of Atherosclerosis. However, they report that the adverse effect of the HOLC category and ideal cardiovascular health was attenuated for African Americans based on neighborhood social environments. Building upon Mujahid et al.’s findings, while we did not consider neighborhood social environments specifically in our models, our finding that the beneficial impacts of living in a yellowlined neighborhood were greater for whites compared with African Americans suggests the importance of understanding more specifically the potentially protective impacts of neighborhood social environment.

### Neighborhood deprivation index as a mediator of race-moderated associations between HOLC scores and allostatic load

Findings reported here suggest that a neighborhood deprivation index (NDI) helped to explain racial differences in the relationship between historical HOLC score and contemporary allostatic load only among African Americans living in formerly yellowlined areas. Specifically, living in yellowlined, but not redlined, areas was positively associated with NDI. We have previously reported findings across many U.S. cities, suggesting that living in a formerly redlined neighborhood was negatively associated with overall neighborhood investment, and that neighborhood investment was negatively associated with obesity, diabetes, and coronary heart disease prevalence (Andrews et al. 2025). Findings reported here bolster existing literature highlighting the lasting impacts of historical redlining in shaping contemporary patterns of neighborhood-level socioeconomic conditions through impairing the social and economic mobility of contemporary residents (Egede et al. 2023; King et al. 2022). We also report that NDI is positively associated with allostatic load, with associations differing by race. These findings are similar to associations previously reported by Merkin et al., who reported that associations between neighborhood socioeconomic status and cumulative biological risk, a measure akin to allostatic load, varied by race and ethnicity (Merkin et al. 2009). Specifically, they reported an inverse association between neighborhood socioeconomic status and cumulative biological risk, after controlling for sociodemographic characteristics. Taken together, these findings underscore the longstanding impacts of historical practices that continue to shape contemporary patterns of socioeconomic conditions relevant to health within the United States.

Strengths of this study include the availability of adequate numbers of African American and white adults in the sample to afford sufficient statistical power to investigate both main and moderating effects in associations between historical HOLC scores and allostatic load, and when considering the role of neighborhood deprivation as a potential mediator. This study also contributes to the growing literature around HOLC scores on contemporary health outcomes. Study limitations include potential selection bias since HANDLS participants were selected from 13 pre-determined neighborhoods within Baltimore, Maryland, and the sampling frame was not stratified by historical HOLC scores. This sampling frame may affect the generalizability of results to the Baltimore area, as the HANDLS frame is representative of the neighborhoods sampled, but not necessarily of the city as a whole.

The variability in the number of observations within the included census tracts, and in some cases, small n’s, may also limit the generalizability of these findings. Further, some study covariates at the individual level were self-reported (e.g. age, race, physical activity), which could contribute to some same-source bias. However, since the key variables of interest (allostatic load, HOLC score, neighborhood socioeconomic indicators) were derived either through clinical measurements (allostatic load), historical redlining maps, or ACS data (neighborhood poverty), the risk of the same source bias substantially affecting the main associations reported here is minimal. Caution should, however, be exercised in attempting to generalize findings to the city of Baltimore, other cities, or geographic areas. For example, insufficient racial representation in formerly green, blue, and redlined areas may hinder our understanding of the relationship between HOLC score and allostatic load (Table 1). Further, compared to the full HANDLS Wave 4 cohort, the analytic sample contains individuals who have lower allostatic load, thus limiting the generalizability of our findings to the broader HANDLS cohort. Nonetheless, while noting the limitations of this dataset, this study underscores the need for continuing research on the legacy of HOLC appraisals and potential health-relevant pathways of effects on the health of current residents.

## Conclusion

The findings reported here join a growing body of literature linking historical policies to contemporary health outcomes. The equitable investment of quality and sustainable resources and support in historically dis- and underinvested communities can mitigate some of the effects of neighborhood contexts on health outcomes. Continued work is needed to further elucidate these complex relationships.

## Figures and Tables

**Table 1.** Distribution of key variables in Wave IV (2013–2017) for the overall study area, and by historically coded Green and Blue lined, yellowlined, and redlined census tracts (CT).

		Green- and Bluelined	Yellowlined		
	Total sample (*N* = 1,013)	CTs (*N* = 321)	CTs (*n* = 573)	Redlined CTs (*n* = 119)	Test of Significance
	Mean (SD)	Mean (SD)	Mean (SD)	Mean (SD)	
	Range	Range	Range	Range	

Allostatic Load	1.95 (1.19)	1.83 (1.15)	2.01 (1.19)	2.01 (1.31)	0.09
	0–6	0–6	0–6	0–6	

	n (%)				

HOLC score		321 (31.69)	573 (56.56)	119 (11.75)	
Age Category					
35–44	198 (11.21)	33 (10.28)	67 (11.69)	15 (12.61)	0.61
45–54	530 (30.01)	95 (29.60)	178 (31.06)	39 (32.77)	
55–64	650 (36.81)	118 (36.76)	219 (38.22)	37 (31.09)	
65–76	388 (21.97)	75 (23.36)	109 (19.02)	28 (23.53)	
Race					
White	306 (30.21)	62 (19.31)	220 (38.39)	24 (20.17)	0.00
Mean Allostatic Load		1.42 (1.14)	2.05 (1.18)	1.88 (1.57)	
African American	707 (69.79)	259 (80.69)	353 (61.61)	95 (79.83)	
Mean Allostatic Load		1.93 (1.13)	1.98 (1.20)	2.04 (1.24)	
Sex					
Female	580 (57.26)	185 (57.63)	336 (58.64)	59 (49.58)	0.19
Male	433 (42.74)	136 (42.37)	237 (41.36)	60 (50.42)	
Physical Activity					
<5 min	321 (31.69)	113 (35.20)	178 (31.06)	30 (25.21)	0.23
5–15 min	210 (20.73)	68 (21.18)	120 (20.94)	22 (18.49)	
15–30 min	238 (23.49)	70 (21.81)	131 (22.86)	37 (31.09)	
30–45 min	111 (10.96)	38 (11.84)	61 (10.65)	12 (10.08)	
>45 min	133 (13.13)	32 (9.97)	83 (14.49)	18 (15.13)	
Smoking Status					
Never Tried	204 (20.14)	83 (25.86)	106 (18.50)	15 (12.61)	0.00
Tried, never used regularly	116 (11.45)	39 (12.15)	66 (11.52)	11 (9.24)	
Former User	260 (25.67)	98 (30.53)	131 (22.86)	31 (26.05)	
Current User	433 (42.74)	101 (31.46)	270 (47.12)	62 (52.10)	
Household Poverty Status					
Above Poverty Threshold	635 (62.69)	230 (71.65)	346 (60.38)	59 (49.58)	0.00
Below Poverty Threshold	378 (37.31)	91 (28.35)	227 (39.62)	60 (50.42)	
Neighborhood Deprivation Index	11.28 (3.19)	9.84 (2.46)	12.23 (2.91)	10.64 (2.46)	0.00

**Table 2.** Results from multilevel models regressing allostatic load on HOLC score controlling for age, race, sex, physical activity, smoking status, and poverty status.

	Model 1		Model 2		Model 3	
			
	B(SE)	*P*	B(SE)	*P*	B(SE)	*P*

Intercept	1.82 (0.16)	<0.01	1.42 (0.22)	0.00	0.64 (0.38)	0.10
*Level 2 (Neighborhood)*						
**HOLC score**						
Green and Bluelined (ref)						
Yellowlined	**0.18 (0.08)**	**0.03**	**0.21 (0.08)**	**0.01**	**0.68 (0.17)**	**0.00**
Redlined	0.18 (0.13)	0.16	0.23 (0.13)	0.08	0.51 (0.28)	0.07
*Level 1 (Individual)*						
HOLC * African American	–	–				
Yellowlined*African American					**−0.62 (0.19)**	**0.00**
Redlined*African American					−0.34 (0.31)	0.28
Race	–	–				
White (ref)	–	–	–	–		
African American	–	–	0.11 (0.08)	0.17	0.56 (0.17)	0.00

* Models 2 and 3 are adjusted for age, race, sex, physical activity, smoking status, and poverty status.

**Table 3.** Tests of direct effects of HOLC score and allostatic load, and tests of measures of neighborhood deprivation Index as a mediating pathway in HANDLS participants (*n* = 1,013).

	Path a: Association between HOLC Score and Mediator		Path b: Association between Mediator and AL		Path c: Association between HOLC Score and AL		Path c′: Association between HOLC Score and AL accounting for Mediator		Indirect Effect		
					
	*β* (SE)	*p*	*β* (SE)	*p*	*β* (SE)	*p*	*β* (SE)	*p*	*β* (SE)		*p*

Yellowlined	**2.84 (0.79)**	**0.00**	**0.12 (0.05)**	**0.01**	**0.68 (0.17)**	**0.00**	0.17 (0.09)	0.07	**Black**	**0.29 (0.14)**	**0.04**
									white	0.33 (0.18)	0.07
Redlined	−2.29 (1.31)	0.08	**0.12 (0.05)**	**0.01**	0.51 (0.28)	0.07	0.24 (0.13)	0.07	Black	−0.06 (0.10)	0.55
									white	−0.27 (0.24)	0.28

Note: All models were adjusted for age category, sex, poverty status, physical activity, and smoking and included the interaction between race and HOLC. The interaction between HOLC score and race was accounted for in each of the models.
